# The Forgotten Beats

**DOI:** 10.1159/nim/adxag001

**Published:** 2026-09-28

**Authors:** Andrei P. Sommer

**Affiliations:** Faculty of Science, ISRA University, Amman, Jordan

**Keywords:** Atrial fibrillation, Photobiomodulation, Mitochondria, ATP synthesis, Coupled oscillators

It is, however, a point of the greatest interest that the maintenance of the plateau of the action potential, which prevents fibrillation, appears to be linked to supplies of high energy phosphate such as adenosine triphosphate.

*J.H. Burn, Pharmacology of Cardiac Failure*

Atrial fibrillation (AF) arises from failure of coordination in cardiomyocyte contraction, a process critically dependent on mitochondrial adenosine triphosphate (ATP) synthesis [1–3]. The sinus node is the conductor of the cardiac cell choir: electrical impulses drive the mechanical contractions, demanding continuous ATP provision. Exposure to stress compromises ATP reserves. The coherent cellular ensemble falls into chaos; in an effort to restore order, the remaining energy is burned – sometimes successfully, sometimes ending in catastrophic events. With depleted energy reservoirs, the sinus node has no chance to reimpose its pace. Stressors, including, but not limited to, alcohol excess, tobacco use, sleep deprivation, and combinations thereof, are known to trigger AF. Air pollution is another determinant [4]. Here, we present a quantum biological rationale, assessed in emergency situations, and reproduced, recommending the use of pulsed intercostal photobiomodulation (PBM) as a noninvasive method to rapidly end AF. The principal irradiation parameters playing a role in PBM are listed in Reference [5].

Physicians in ancient Egypt were the first to establish the importance of the pulse and its relationship with the contractions of the heart. Anomalies of cardiac rhythm – what we today call arrhythmia – were recorded in the medical papyri with a poetic precision that continues to provoke: they were called the *forgotten beats* [6]. *Forgotten* instead of *lost* or *missing* implies: the heart knows the program, but as it happens with fatigue, it forgets its task. This linguistic picture, drawn more than three millennia ago, frames arrhythmia as a transient phenomenon. Imagine an orchestra whose musicians know their repertoire but fatigued after a long journey, the drummer forgets his entry and skips the beat. The information is intact; the energy to act on it is compromised. In 2012, upon an exhausting return by train, subsequent to laboratory work carried out in long night shifts, the author experienced for the first time the full spectrum of AF – and treated himself with pulsed red to near-infrared (R-NIR) light. Decades before the molecular era, Burn had reported that exogenous ATP arrests AF and its removal reinstates it [7] – an observation whose mechanistic basis is now accounted for by intracellular ATP restitution via R-NIR PBM.

The understanding of the second mechanism of the proposed intervention was inspired by a night walk in a South Indian jungle at the foot of the Velliangiri Mountains. Initially asynchronous, the flashes in a tree invaded by a colony of fireflies gradually converged into full synchrony. Periodically, the tree went dark, only to light up a moment later. How did the insects achieve this? Arthur Winfree modeled synchronization in populations of coupled biological oscillators, such as flashing fireflies or chorusing crickets [8]. He derived a pacemaker model from this analysis. The analogy to the heart is direct: if a fraction of an ensemble of coupled oscillators can entrain the whole, then pulsed light delivered to the heart at the personalized resting heart frequency should be capable of imposing rhythmic order on a chaotically beating cardiomyocyte ensemble – provided ATP levels suffice to drive the contractions.

Earlier, we showed that in oxidatively stressed cells, where mitochondria switch from generating ATP to the production of reactive oxygen species (ROS), the process can be reversed by irradiation with monochromatic light (lasers, LEDs), preferably in the R-NIR spectrum [9]. The glue-like nature of nanoscopic interfacial water layers formed on hydrophilic surfaces facilitates the transient capture of mitochondrial ROS (superoxide, O_2_•^−^), whose negative charge reinforces the electrostatic ordering of water molecules within the film, further increasing the viscosity beyond its already elevated baseline, thereby impeding the rotation of the mitochondrial nanoturbine (ATP-synthase) [10]. The normal speed of rotation is approximately 9,000 rpm. In analogy to laboratory experiments, R-NIR light instantly restores the rotation speed by reducing the elevated interfacial water viscosity ([11] and references therein) – a purely physical mechanism, as opposed to a complex biochemical one.

A second physical mechanism, most likely shaped by evolution on a primordial Earth, protects the organelles from an overdrive of the nanomotor – a potentially disastrous effect of excessive light exposure – secured via an intrinsic quantum biological field-effect transistor (FET), with its three components *emitter*, *drain*, and *gate*, embodied by cytochrome c, cytochrome c oxidase, and an interfacial water layer, separating the proximal enzymes, respectively [10, 12]. The probability for electrons tunnelling across the gate is controlled by R-NIR light: intensities exceeding a biocompatible threshold, result in a widening of the gate, concomitant with attenuation of the electron current between the enzymes – thereby reducing ATP output and providing a natural brake on mitochondrial overdrive. The expansion of nanoscopic interfacial water layers irradiated with R-NIR light is confirmed experimentally [13].

Electrons transferred from cytochrome c to cytochrome c oxidase serve as fuel to pump protons across the mitochondrial membrane – protons that, as Peter Mitchell proved, drive ATP synthase to synthesize ATP. The comparison with the effect of cyanide is instructive: blockage of the electron current between the enzymes causes the proton gradient across the mitochondrial membrane to collapse, ATP synthesis to cease, with death occurring within minutes, depending on the dose. Too intense R-NIR irradiation manifests itself similarly, albeit reversibly and partially, via suppression of the electron current between cytochrome c and cytochrome c oxidase.

An example demonstrating that overexposure to R-NIR light is sensed by cells as stress is the treatment of herpes labialis with red laser light at an intensity of 20 kWm^−2^ [14]. Under optimal atmospheric conditions, the maximum terrestrial solar intensity reaches 1 kWm^-2^. It is perhaps not a coincidence that virtually all tissues harboring mitochondria respond to R-NIR light of this intensity by producing ATP – both in vitro and in vivo – suggesting that early bacteria, precursors of mitochondria, were adapted to harvest solar photons for ATP synthesis. Infections with herpes simplex virus type 1, the virus responsible for herpes labialis, are known to trigger neuroinflammatory cascades implicated in neurological conditions such as Alzheimer’s disease [15, 16]. Untreated, symptoms last 1–2 weeks. Topical acyclovir may reduce this duration by 1 day. Irradiation of the affected area at the onset of first symptoms with 650 nm laser light at 20 kWm^−2^ for 5 min, twice daily for 2 consecutive days, prevented an outbreak – no inflammation, no pain, no bleeding. The treatment was reproduced on several occasions. Results were identical. What is the root cause of the antiviral effect of “20 suns”?

Macrophages use ROS to combat bacteria and viruses. Their work is even supported by R‐NIR light. But since ROS is for macrophages a red flag, it can be safely assumed that the virus is kept at bay by mitochondrial ROS, triggered by the high light intensity. Possible involvement of herpes simplex virus type 1 in neurological conditions and the rapid recovery from herpes labialis are sufficient motivation for further research efforts with R-NIR light at ATP suppressive intensities [14]. Additional clarity emerges from the consideration of mitochondrial function in the transition of macrophages from the clean-up phenotype (M1) to the reparative phenotype (M2), in particular in ischemic diseases of the heart. In a widely cited study [17], Cai et al. present exciting results indicating that cardiac injury and impaired scar formation are promoted by mitochondrial dysfunction in macrophages – a picture turning mitochondrial function into a potential therapeutic target. For this, Cai et al. discriminate between the proinflammatory phenotype (M1), predominantly operating in the glycolytic mode, using excessive mitochondrial ROS, and the reparative phenotype (M2), with ATP produced via oxidative phosphorylation in mitochondria. Understanding the interplay between M1 and M2, on the one hand, and the associated mutually exclusive interplay between ATP and ROS, on the other hand, is the key for modulating the resolution of the inflammation and healing of the tissue by light. In eliminating herpes simplex virus type 1, the intense laser light was pointed at one spot (suppressing ATP synthesis and promoting ROS release) – during the AF episodes, the laser probe was constantly repositioned, a shift toward ATP generation via oxidative phosphorylation. Extension of PBM from the treatment of AF to ischemic conditions of the heart receives support from the good transmission of blood for R-NIR light as well as from its well-known vasodilatory effect, concomitant with vascular permeabilization [18], which facilitates the extravasation of monocytes into the lesion zone – altogether strong aspects in support of a cardioprotective role of light-modulated mitochondrial function. Instead of, or complementary to, administering ROS scavengers in M1, the same ATP-restoring PBM regime used in AF naturally recommends itself to downregulate ROS and upregulate ATP synthesis, favoring the M1-to-M2 transition. Here too, early intervention is reasonable.

So far, the picture summarizes our understanding of interaction modalities between R-NIR light and mitochondria. This picture puts us into the position to design a potent AF therapy. External replenishment of the cardiac ATP pool demands a tissue window with minimal optical absorption at therapeutically relevant intensities – the intercostal space. From 2012 to 2025, four AF episodes were treated by R-NIR light, carried out with the laser constellation described in Figure 1. Heart rate variability (HRV) was derived from photoplethysmographic recordings. In the episode reported here, the transition from rapid irregular beats typical for AF (Fig. 2) to a stable pattern without gaps (Fig. 3) was reached within 20 min of irradiation. The rapid AF resolution experienced in all episodes opens an avenue to a patient-friendly rescue therapy, justifying further research. The power of the lasers employed renders wavelength-appropriate eye protection mandatory for both operator and subject, a requirement that applies to any future device iteration.

**Fig. 1.**
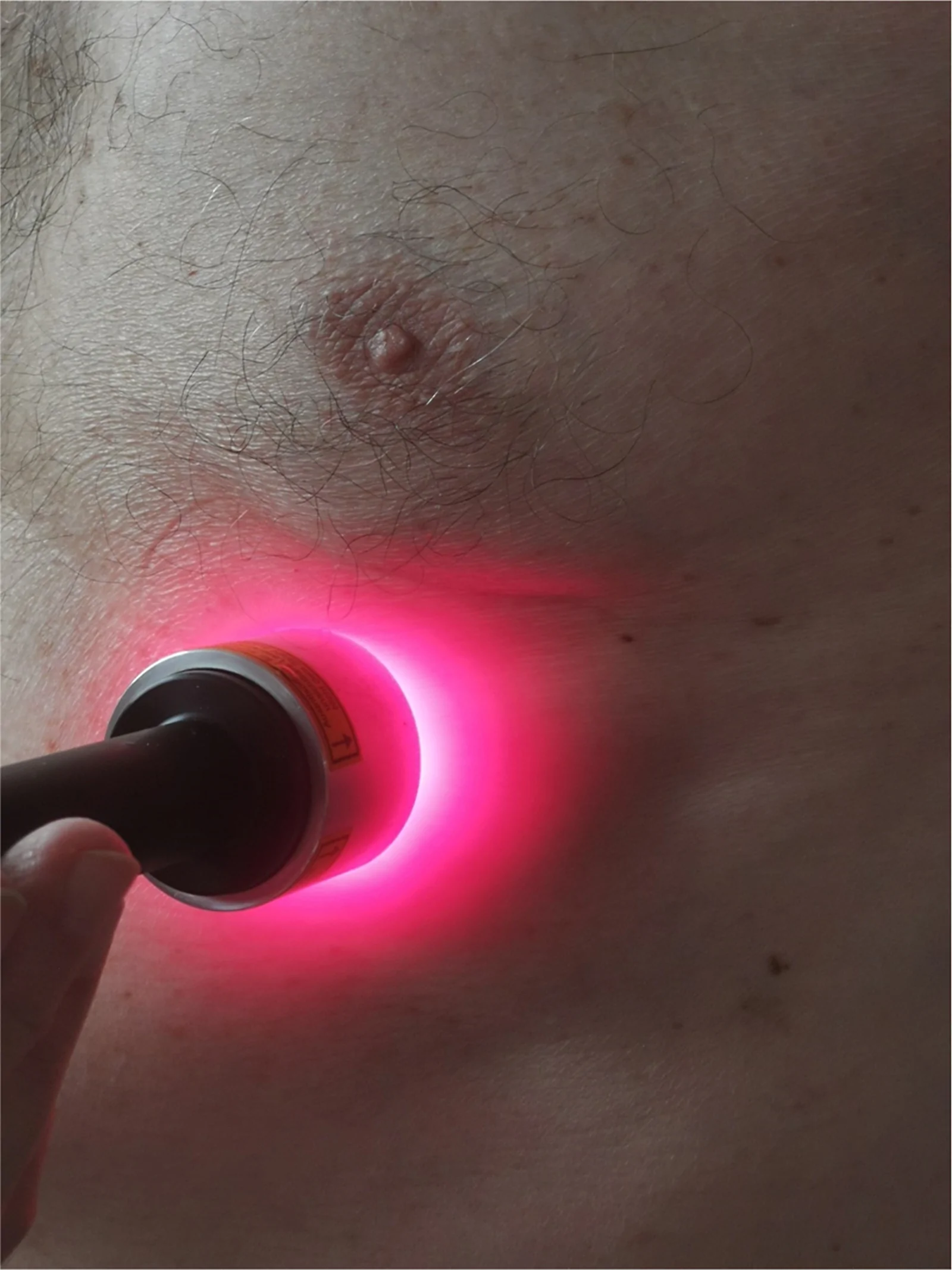
Self-treatment during AF. The laser power is high enough to reach the heart but below skin-burning levels. To prevent overdoses and to cover larger cardiac areas with photons, the laser probe was constantly repositioned during the intervention. For better light transmission, the thorax was held in full inspiration to maximize intercostal spacing. R-NIR prototype for intercostal PBM: aluminum head holding six 660 nm laser diodes and one 780 nm laser diode, each delivering 80 mW (measured surface irradiance: 50 kWm^−2^), is placed over the left precordium. Irradiation of the underlying myocardium is carried out at 60 pulses per minute, matching the subject’s resting heart rate, for 20 min. The duty cycle was set to 90%, corresponding to a periodic modulation with 0.9 s of irradiation followed by 0.1 s off, stimulating a heartbeat pattern of nearly 1 Hz, at near-continuous photon delivery. During the entire intervention, skin temperature increase was moderate and easily tolerated; the felt warmth was ascribed to gradual heating of the aluminum probe rather than to a photothermal effect in the skin, as confirmed by persistence of warmth after the lasers were switched off. Beam divergence, tissue absorption, and scattering each contribute independently to some attenuation of the initial irradiance across the intercostal path, with a photon flux estimated to reach the myocardium at biostimulatory levels. Heat raises superficial skin blood flow via vasodilation and thus hemoglobin absorption. Cooling suppresses this, preserving photon flux without thermal skin damage. An advanced model would incorporate a laser probe with active cooling.

**Fig. 2.**
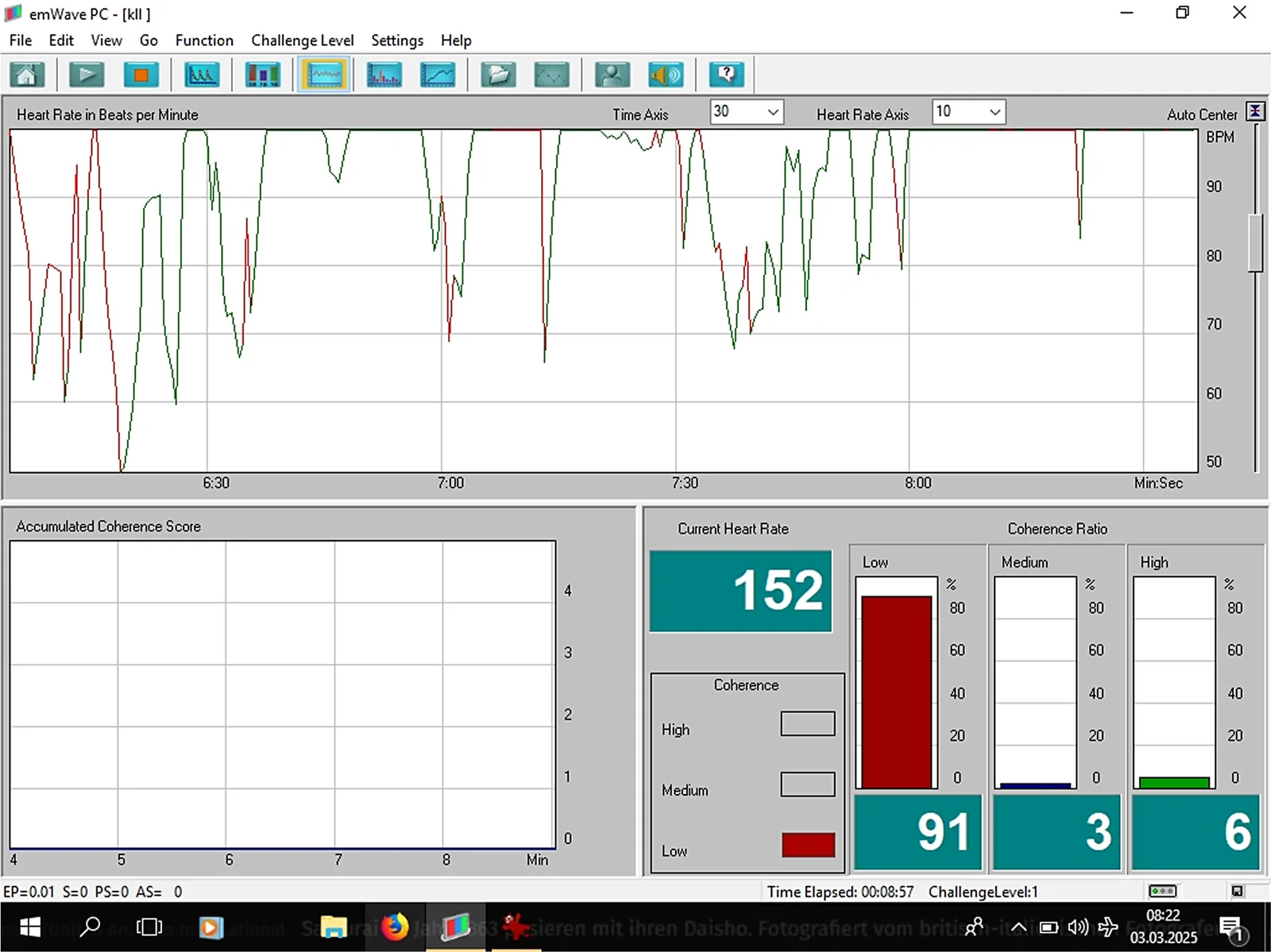
Photoplethysmographic recording of typical fibrillation event prior to intercostal PBM, illustrating a chaotic pulse pattern with manifest beat gaps, consistent with AF and reflecting the forgotten beats experienced during the episode (recording duration 8 min 57 s) (recorded using the HeartMath emWave system, Boulder Creek, CA 95006, USA).

**Fig. 3.**
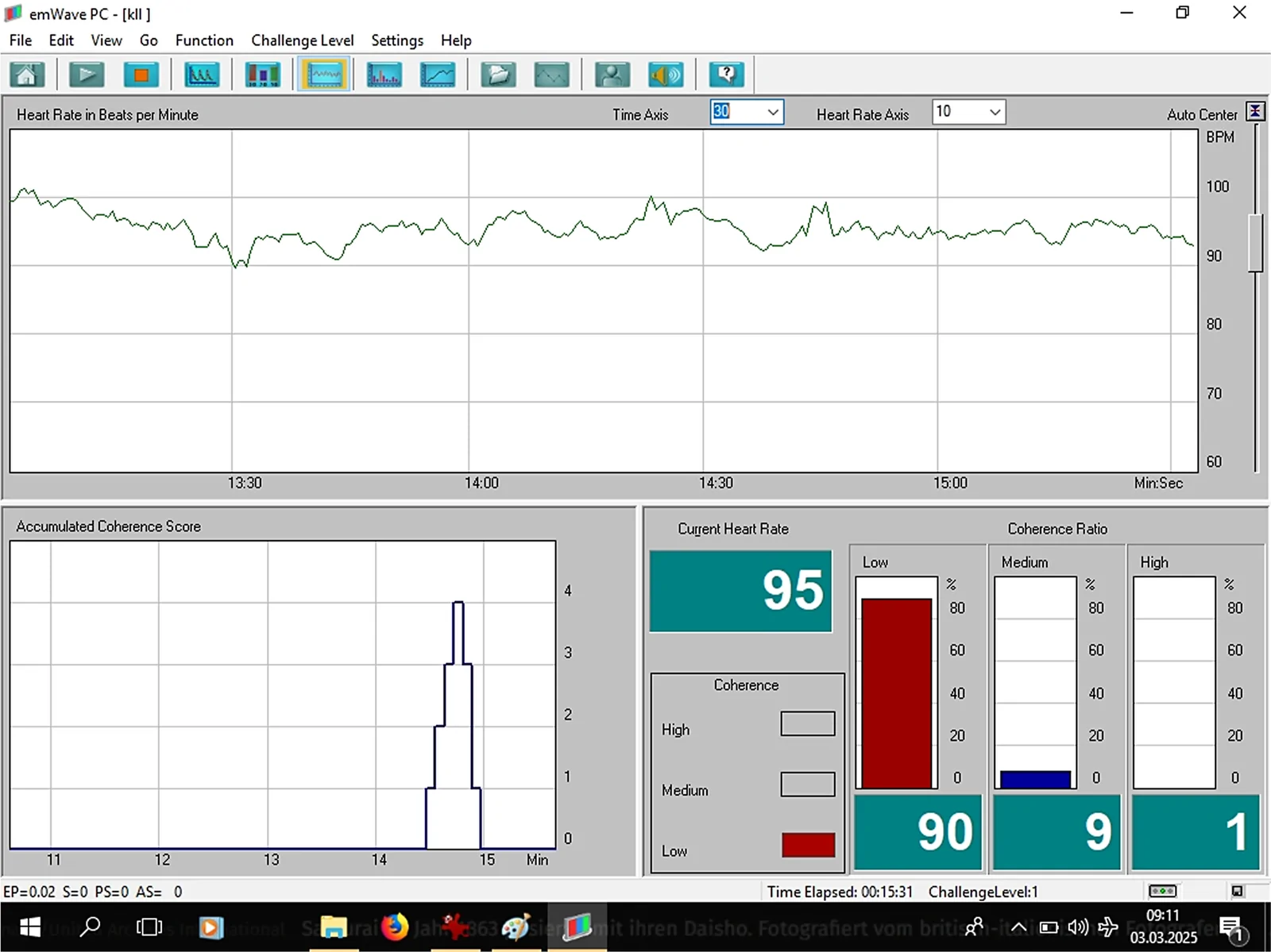
Restored pulse pattern following 20 min of intercostal irradiation (recording duration 15 min 31 s). Data acquired as in Figure 2. Transition to a regular beat pattern began within approximately 2 min of irradiation onset. The laser intervention took place in the time between the recordings of Figures 2 and 3. No adverse effects were observed.

The gap between the low imposed pulse frequency and the high AF frequency suggests, per Winfree’s coupled-oscillator theory, suboptimal entrainment. In contrast, the observed rapid cardioversion may indicate that replenishment of ATP is the primary restorative mechanism. Starting with light pulses at the AF frequency and ramping down toward resting heart rate with real-time cardiac feedback would allow these two mechanisms to be distinguished and may improve cardioversion efficiency in future clinical studies. One observation merits attention: based on four symptomatic AF episodes, all triggered by the classic holiday heart triad [19], early intervention is essential. Prompt irradiation may prevent progressive mitochondrial dysfunction and ROS buildup that would otherwise perpetuate the arrhythmia: AF begets AF [20] – the longer AF runs, the more the atrial myocardium undergoes changes that make AF increasingly self-sustaining and harder to cardiovert.

Translation of this proof of concept into dedicated precordial devices combining heart-rate-variability-adapted PBM for use at home and in ambulances is promising, subject to confirmation by controlled clinical trials. R-NIR modulation of mitochondrial function was shown to reduce brain injury following cardiac arrest in animals [21]. Further, a chemo-optical strategy has been proposed for targeting Alzheimer’s disease [18, 22], suggesting that beneficial effects observed in the heart extend naturally to the neuroimmune interface. The forgotten beats of the ancient papyri may thus find their contemporary answer not in pharmacology but in light.

## Conflict of Interest Statement

The author has no conflicts of interest to declare.

## Funding Sources

This study was not supported by any sponsor or funder.

## Author Contributions

Andrei P. Sommer is the sole author. The author confirms sole responsibility for the study conception and design, data collection, analysis and interpretation of results, and manuscript preparation.
